# Authenticity and drug resistance in a panel of acute lymphoblastic leukaemia cell lines

**DOI:** 10.1038/sj.bjc.6603447

**Published:** 2006-11-21

**Authors:** A H Beesley, M-L Palmer, J Ford, R E Weller, A J Cummings, J R Freitas, M J Firth, K U Perera, N H de Klerk, U R Kees

**Affiliations:** 1 Division of Children's Leukaemia and Cancer Research, Telethon Institute for Child Health Research, and Centre for Child Health Research, University of Western Australia, Perth, Australia; 2 Curtin University of Technology School of Pharmacy, Perth, Western Australia; 3 Division of Biostatistics and Genetic Epidemiology, Telethon Institute for Child Health Research, and Centre for Child Health Research, University of Western Australia, Perth, Australia

**Keywords:** acute lymphoblastic leukaemia, drug resistance, cell line, methotrexate, glucocorticoid, fingerprint

## Abstract

Cell lines are important models for drug resistance in acute lymphoblastic leukaemia (ALL), but are often criticised as being unrepresentative of primary disease. There are also doubts regarding the authenticity of many lines. We have characterised a panel of ALL cell lines for growth and drug resistance and compared data with that published for primary patient specimens. In contrast to the convention that cell lines are highly proliferative, those established in our laboratory grow at rates similar to estimates of leukaemic cells *in vivo* (doubling time 53–442 h). Authenticity was confirmed by genetic fingerprinting, which also demonstrated the potential stability of long-term cultures. *In vitro* glucocorticoid resistance correlated well with that measured *ex vivo*, but all lines were significantly more sensitive to vincristine than primary specimens. Sensitivity to methotrexate was inversely correlated to that of glucocorticoids and L-asparaginase, indicating possible reciprocity in resistance mechanisms. A cell line identified as highly methotrexate resistant (IC_50_ >8000-fold higher than other lines) was derived from a patient receiving escalating doses of the drug, indicating *in vivo* selection of resistance as a cause of relapse. Many of these lines are suitable as models to study naturally occurring resistance phenotypes in paediatric ALL.

In children with acute lymphoblastic leukaemia (ALL) cellular drug sensitivity is a major component of clinical outcome. This is true not only for relapsed ALL (Klumper *et al*, 1995) where *in vivo* selection of resistant clones occurs during therapy, but also in those newly diagnosed with the disease (Kaspers *et al*, 1997; Pieters *et al*, 1998). Much of our knowledge of the resistance phenotypes in ALL has been derived using isolated bone marrow specimens studied in short-term culture using the 3-(4,5-dimethylthiazol-2-yl)-2,5-diphenyltetrazolium bromide (MTT) assay. However, application of this *ex vivo* approach for functional studies or drug screening is limited by both the availability of patient material and the short period of survival of these cells in culture. Furthermore, this approach cannot be used to measure methotrexate (MTX) resistance in such specimens because the high rate of spontaneous cell death releases nucleosides in sufficient quantity to prevent MTX cytotoxicity (Pieters *et al*, 1997). Investigation of resistance mechanisms and evaluation of novel drug-leads invariably requires the use of immortalised cell lines, but the extent to which these cells retain features of the original disease *in vivo* is a matter of some debate (Kamb, 2005), a problem exemplified by the typically high growth rates of continuous cultures (Masters, 2000). Added to this is concern over the alarming frequency with which cultures have been found retrospectively to be infected with mycoplasma or cross-contaminated with other cell lines (so-called ‘false’ lines) (Masters, 2000; Drexler *et al*, 2003). This has led to repeated calls for the extensive characterisation and validation of authenticity of such cell lines (Drexler and Matsuo, 1999; Masters *et al*, 2001; Drexler *et al*, 2002, 2003).

Over the past 20 years our laboratory has developed a panel of paediatric ALL cell lines that have been grown in the absence of drug selection. Previously, we have shown, using a subset of this panel, that these cultures retain critical immunophenotypic and molecular features of the primary disease (Kees *et al*, 2003). Here, we have validated the genetic identity of these lines and have systematically studied their drug resistance and growth profiles to assess the degree to which they reflect the phenotype of primary ALL patient specimens.

## MATERIALS AND METHODS

### Cell lines

PER cell lines were derived from paediatric ALL bone marrow specimens as described previously (Kees *et al*, 1987). Patients were diagnosed and treated at the Princess Margaret Hospital for Children, Perth, Western Australia, and informed consent was obtained from parents, patients, or both as deemed appropriate. Several of the cell lines have been described previously (Kees, 1987; Kees *et al*, 1987, 1989a, 1989b, 1990, 1995, 2003; Kennedy *et al*, 1991; Whitman *et al*, 2001). CCRF-HSB2 (HSB2) cells were obtained from the American Type Culture Collection; CCRF-CEM (CEM) from the Children's Cancer Institute Australia for Medical Research, Sydney; JURKAT from the Basel Institute for Immunology, Switzerland; ALL-SIL from MRC Laboratory of Molecular Biology, Cambridge, UK; MOLT4 from the German Cancer Research Center, Heidelberg, Germany; DU.528 from the Division of Cancer Biology, Telethon Institute for Child Health Research, Perth. Cell lines were grown in RPMI-1640 supplemented with 2 mM L-glutamine, 10 nM 2-mercaptoethanol and 10–20% heat-inactivated foetal calf serum. All PER cell line media contained additional non-essential amino acids and pyruvate, whereas 300 U/ml interleukin-2 is required for growth of PER-427 and PER-487 (Kees *et al*, 2003). Cell lines were cultured in the absence of antibiotics; testing for mycoplasma was routinely performed by PCR, and immunophenotyping performed by indirect immunofluorescence and flow cytometry (Kees *et al*, 1987, 2003). Doubling times were determined using the MTT assay (described below) and extensive laboratory records. DNA fingerprinting of matched cell lines and patient specimens was performed by Genetic Technologies Corporation Pty Ltd (Melbourne, Australia) using the AmpF/short tandem repeat (STR) Identifiler kit which co-amplifies 15 STR loci and the sex determination locus Amelogenin. Allelic profiles were assessed by calculating the percentage of alleles at the 16 loci present in cell lines that were also co-identified in the corresponding patient specimen (Supplementary Table 1).

### *In vitro* drug resistance

Resistance was tested using the MTT assay (Alley *et al*, 1988). Cells in exponential growth phase were counted by trypan blue exclusion and seeded in fresh media at a density of 5 × 10^5^–1.5 × 10^6^ cells ml^−1^ in a 96-well plate in the presence or absence of each drug. Drugs were serially diluted in fresh media, with each drug concentration tested in triplicate. Culture plates were incubated for 4 days at 37°C before the addition of 10 *μ*l of filter-sterilised MTT (5 mg ml^−1^). Plates were re-incubated for 6 h before addition of 100 *μ*l of acidified isopropyl alcohol solution to dissolve formazan crystals and measurement of absorbance at 590 nm. Testing was performed using two-fold step dilutions of the following drug ranges: cytosine arabinoside (ARA-C; Pharmacia Pty Ltd, NSW, Australia) 0.3 pg ml^−1^–2.5 *μ*g ml^−1^; dexamethasone (DEX; Mayne Pharma Pty Ltd, VIC, Australia) 30 pg ml^−1^–250*μ*g ml^−1^; methylprednisolone (MPRED; David Bull Laboratories, VIC, Australia) 30 pg ml^−1^–250 *μ*g ml^−1^; 6-thioguanine (6TG; GlaxoSmithKline Australia Pty Ltd, VIC, Australia) 12 pg ml^−1^–100 *μ*g ml^−1^; 6-mercaptopurine (6MP; GlaxoSmithKline) 60 pg ml^−1^–500 *μ*g ml^−1^; daunorubicin (DNR; Pharmacia) 0.2 pg ml^−1^–2 *μ*g ml^−1^; doxorubicin (DOX; Mayne Pharma Pty Ltd) 1 pg ml^−1^–8*μ*g ml^−1^; L-asparaginase (ASP; Kyowa Hakko Kogyo Co. Ltd, Tokyo) 2.4 × 10^−6^–20 IU ml^−1^; vincristine (VCR; Pharmacia) 23 fg ml^−1^–195 ng ml^−1^; methotrexate (MTX; David Bull Laboratories) 60 pg ml^−1^–500 *μ*g ml^−1^. The IC_50_ (drug concentration that inhibits cell growth by 50%) was used as the measure of drug resistance. Data represent the average of 2–6 separate experiments and linearity was checked between viable cell number and optical density for each cell line. Where 50% cytotoxicity was not achieved by even the highest dose, the IC_50_ was recorded as double the highest concentration tested.

### Comparison of *in vitro* and *ex vivo* resistance profiles

Data from multiple studies (Pieters *et al*, 1990, 1991, 1998; Klumper *et al*, 1995; Duyn *et al*, 1999; Styczynski *et al*, 2000, 2002, 2005; Zwaan *et al*, 2000; Mihal *et al*, 2004; Fine *et al*, 2005; Kaspers *et al*, 2005; Steinbach *et al*, 2005) were combined to determine an absolute minimum and maximum IC_50_ range for bone marrow specimens obtained from paediatric ALL patients at the time of diagnosis (PD) or relapse (PR). Data reported in these studies as LC_50_ values are, for simplicity, referred to here in terms of IC_50_. Eligible studies satisfied the following criteria: (i) drug sensitivity was tested in primary ALL specimens using the MTT assay; (ii) drug incubation was for 3–5 days with no exposure to other agents before testing; (iii) resistance was reported using IC_50_ or LC_50_ values; (iv) patients were under 19 years of age and were divided into diagnosis and relapse cohorts. In the two cases where patients were stratified by immunophenotype (Pieters *et al*, 1998; Kaspers *et al*, 2005), data from T- and B-lineage specimens were combined before analysis. Data for MPRED were available from only one study (Styczynski *et al*, 2002), Whilst MTX sensitivity cannot be measured in primary specimens using the MTT assay (Pieters *et al*, 1997).

### Statistical analysis

All comparisons were performed on Log_2_ IC_50_ data; the Epstein–Barr virus (EBV)-transformed cell line PER-607 was not included in any of the analyses. Significant differences in IC_50_ values between drugs (in molarity) were determined using the nonparametric Wilcoxon-matched pairs signed ranks test. All other differences were assessed using the Mann–Whitney *U*-test. Spearman's correlations were used to assess relationships between drug profiles (IC_50_ values). Delta IC_50_ profiles for MPRED, DEX, ASP and MTX were calculated by subtracting median Log_2_ IC_50_ values (T-ALL cell lines, *n*=15) from the Log_2_ IC_50_ score for each cell line.

## RESULTS

### Characterisation of cell lines

The 17 cell lines developed in our laboratory are listed in Table 1. The panel comprises nine T-ALL and seven B-lineage ALL lines derived from children at different stages of disease (diagnosis or relapse), and one EBV-transformed cell line (PER-607) which was originally derived from the diagnostic specimen of a patient that expressed both T- and B-cell markers. The cell line demonstrated a B-lineage immunophenotype and the presence of EBV was confirmed by PCR. DNA fingerprinting verified the genetic identity of the cell lines, with 13 out of 17 lines showing 100% allelic concordance with original patient specimens across 16 genomic loci (Table 1 and Supplementary Table 1). The minor allelic variations we observed in four lines (88–97% concordant) are consistent with the genetic drift associated with cancer cells grown in culture over extended periods (Masters *et al*, 2001). The primary patient specimen for PER-117 demonstrated additional (tertiary) alleles in low abundance at several loci (6 out of 16 loci), which were not present in the DNA from PER-117 (Supplementary Table 1). This specimen was obtained from a patient who relapsed 2 months after receiving a bone marrow transplant from his brother; the minor alleles are indicative of the presence of residual donor cells in the marrow aspirate at relapse.

### Cell line drug resistance profiles

The cell lines in Table 1 and six additional T-ALL cell lines obtained from external sources, were tested for their sensitivity to the 10 drugs most commonly used in the treatment of paediatric ALL. The IC_50_ values for each cell line are listed in Table 2 along with their doubling time for growth in culture. The experimental protocol (i.e. measurement by MTT assay after a 4-day drug incubation) was modelled on the approach successfully used to assess drug resistance in primary ALL bone marrow specimens *ex vivo* (Klumper *et al*, 1995; Kaspers *et al*, 1997, 2005). For most drugs, sensitivities ranged over several orders of magnitude. The EBV-transformed cell line PER-607 had relatively high IC_50_ values for most of the drugs tested, although these were still within the range demonstrated for ALL lines. Although included here for general interest, the drug resistance profile of this cell line was not included in subsequent analyses.

**Figure 1:**
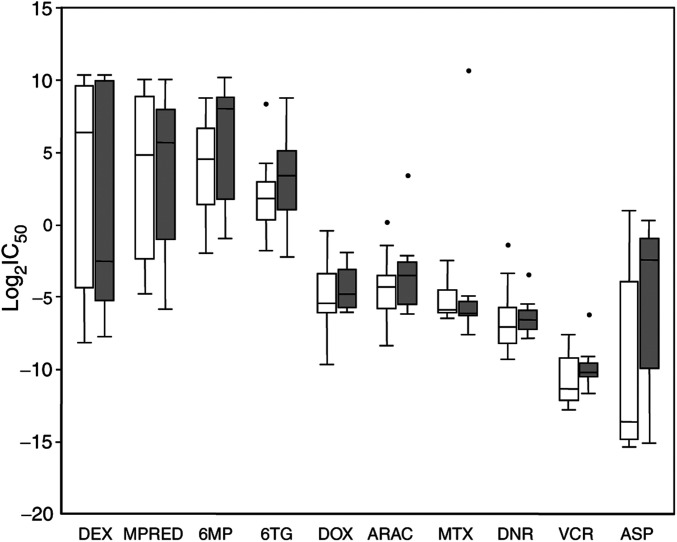
Drug resistance profile of ALL cell lines. IC_50_ values for T-ALL (open, *n*=15) *vs* B-lineage ALL (shaded, *n*=7) cell lines. Boxes indicate medians and inter-quartile range, whiskers indicate 10th and 90th percentiles and dots indicate outliers; values are calculated as log_2_ molarity (*μ*M) for all drugs, except ASP which is given as log_2_ IU ml^−1^.

The resistance profiles of the 15 T-ALL and seven B-lineage ALL cell lines are shown graphically in Figure 1. Among T-ALL lines (open boxes), greatest resistance was to the steroids (DEX and MPRED) and thiopurines (6MP and 6TG) with median IC_50_ values several orders of magnitude higher than the other drugs; greatest sensitivity was to VCR and ASP. A similar drug profile was observed in the B-lineage cell lines (Figure 1, shaded boxes). The parental CCRF-CEM cell line is known to represent a mixed population of sensitive and resistant clones (Medh *et al*, 2003); IC_50_ values for DEX and ASP for this cell line (Table 2) were much higher than have been reported elsewhere (Martin-Aragon *et al*, 2000; Catts *et al*, 2001; van der Heijden *et al*, 2004), indicating that our CEM represents a resistant sub-clone that has grown out during culture.

Although there were considerable differences in medians between the T- and B-lineage ALL cell lines for some drugs, particularly DEX and ASP, these did not reach statistical significance owing to the considerable variation observed within each lineage (Mann–Whitney *U*-test, *P*>0.05). Previous work in primary specimens has indicated that T-ALL specimens are more resistant than precursor B-lineage to several front-line drugs, especially ASP, VCR and glucocorticoids (Pieters *et al*, 1998). Although the trend for DEX in our data is in agreement with these findings, for the other drugs, particularly ASP, this is not the case. Larger *in vitro* studies are required to confirm these findings.

Daunorubicin was approximately three times more potent than DOX in both lineages (Wilcoxon-matched pairs test: T-ALL, *P*<0.001; B-lineage ALL, *P*=0.018; T and B lineages combined, *P*<0.0001), whereas 6TG was 6–24 times more cytotoxic than 6MP (Wilcoxon-matched pairs test: T-ALL, *P*<0.005; B-lineage ALL, *P*<0.05; T and B lineages combined, *P*<0.0005). These observations are consistent with previous reports of the *in vitro* cytotoxicity of these agents (Adamson *et al*, 1994; Klumper *et al*, 1995; Pieters *et al*, 1998; Kaspers *et al*, 2005). No significant differences in cytotoxicity were observed between DEX and MPRED (*P*>0.05).

An increase in resistance to many front-line drugs, particularly the glucocorticoids, has been reported at the time of ALL relapse (Klumper *et al*, 1995; Rots *et al*, 2000), the phenomenon being most pronounced in those with pre-B-ALL (Kaspers *et al*, 2005). In the present study, there was no significant difference in drug sensitivity between cell lines derived from diagnosis or relapse specimens, but there was a trend towards increased steroid resistance in B-ALL lines derived from relapse patient (Table 1).

### Comparison of *in vitro* and *in vivo* drug resistance

**Figure 2:**
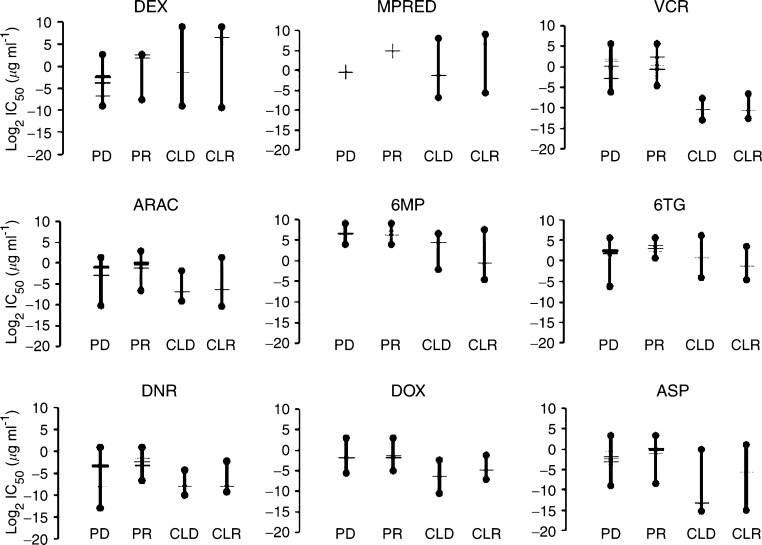
Comparison of resistance profiles in patients and cell lines. Data indicate total IC_50_ ranges determined from published studies of diagnosis (PD) and relapse (PR) patient specimens, and from cell lines derived from diagnosis (CLD) and relapse (CLR) specimens in the present study. Median values from individual studies are indicated as tick marks. Median values from the single study of MPRED resistance in patient specimens are indicated as crosses. Data represent combined T and B lineages; values are *μ*g ml^−1^ for all drugs, except ASP which is given as IU ml^−1^.

To assess how the spectrum of drug resistance observed in the cell line panel related to levels of resistance found in patients, we compared our data with that published from *ex vivo* studies of primary paediatric ALL specimens. The studies included in this analysis are shown in Table 3 and were selected using strict criteria for experimental design, both to minimise variations caused by laboratory handling and to facilitate comparison with data obtained in the present study (see Materials and Methods). As most of the published data were not stratified for immunophenotype, data for T- and B-lineage cell lines were combined for this analysis. The data are compared with cell line resistance profiles in Figure 2, with the medians from the multiple studies indicated by individual tick marks. Studies of primary ALL specimens have consistently indicated an increase in median glucocorticoid resistance at relapse (Figure 2, PR), yet some patients are highly resistant to these agents even at the time of first diagnosis (Figure 2, PD), with the maximum reported range for DEX being similar in both cases. For the other compounds in Figure 2, diagnosis and relapse resistance profiles overlap significantly reiterating the particular importance of steroid resistance in relapsing patients. Among the cell lines (CLD/CLR), some were particularly resistant to DEX, exceeding LC_50_ values reported for even the most resistant relapse patient (Figure 2 and Table 2). However, the highest dose tested in the patient studies was 6 *μ*g ml^−1^, many times lower than the maximum dose used in the present study (250 *μ*g ml^−1^) and so the reported LC_50_ range for patients at relapse is likely to be an underestimate.

All cell lines, without exception, were significantly more sensitive to VCR than patient specimens. Lines were also more sensitive to ASP, 6MP and DOX than primary specimens (primarily owing to lower values in T-ALL cell lines, see Figure 1), although these differences were not as extreme. Data for MTX resistance measured using the MTT assay in primary specimens are not available. Methotrexate sensitivities have been successfully compared in primary specimens using *in situ* inhibition of thymidylate synthetase (Rots *et al*, 2000), but the data are not comparable to the growth inhibition studies performed in the present study. However, peak plasma concentrations after a 20 mg m^−2^ oral dose range from 0.1 to 1.4 *μ*g ml^−1^ (Balis *et al*, 1998), and in comparison to this, all cell lines with the exception of PER-145 were relatively sensitive (Table 2). PER-145 was extraordinarily resistant to MTX, having an IC_50_ >8000 times higher than even the next most resistant cell line.

### Cross-resistance between drugs

**Figure 3:**
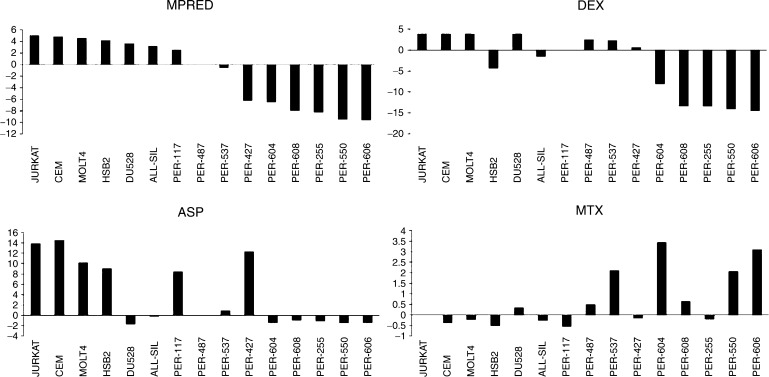
Resistance profiles of MPRED, DEX, ASP and MTX in T-ALL cell lines. Delta IC_50_ scores (Log_2_ IC_50_ – median Log_2_ IC_50_) were calculated for each drug and cell lines plotted from left to right according to their Delta IC_50_ rank for MPRED.

Spearman's correlations were used to identify drugs with similar profiles across the T-ALL cell line panel, which represents a larger and more homogenous group than the B-lineage cell lines. IC_50_ scores for drugs with a similar mechanism of action (Table 4, boxed) were highly correlated to each other (DEX *vs* MPRED, DNR *vs* DOX and 6MP *vs* 6TG, all *P*<0.001), demonstrating that this is a valid approach for the identification of potential cross-resistance between compounds, although it should be emphasised that these relationships were not directly tested in the present study. DEX showed significant correlation (bold values, *P*<0.05) to all drugs, except MTX and the thiopurines; MPRED mirrored this pattern, particularly in regard to ASP, DNR and MTX, the latter showing an inverse relationship as it did with DEX. The relationship between MPRED, DEX, ASP and MTX in the T-ALL cell lines is demonstrated graphically in Figure 3, which displays the resistance spectrum of the panel for each of the four drugs. The Delta IC_50_ score used in this analysis essentially ranks the cell lines for their resistance in comparison to the population median (positive scores more resistant than the median, negative scores more sensitive than the median). Cell lines resistant to MPRED, DEX and ASP were generally the most sensitive to MTX and *vice versa* (*P*<0.05 for negative correlation between MTX and ASP, Table 4). Resistance profiles to the anthracyclines (DNR and DOX), ARA-C and VCR were all significantly correlated to each other in T-ALL cell lines (Table 4, all *P*⩽0.005), indicating cross-resistance.

## DISCUSSION

A frequent criticism of cancer cell lines is that they are unrepresentative of the primary disease as they originate from highly proliferative cell populations that are particularly amenable for growth in culture (Masters, 2000). This is exemplified by the six cell lines included in this study that were obtained from external sources, all of which grow quickly and are easy to maintain (Table 2). In contrast, many of our own cell lines are slow growing and were extremely difficult to establish. Notably, our panel represents an unselected cohort of patients, with half of the lines originating from diagnostic specimens. The doubling time of non-leukaemic pre-B cells measured *ex vivo* is ∼65–90 h, whereas leukaemic pre-B cells are thought to be more heterogeneous, ranging from 25 to 240 h (Hirt *et al*, 1992; Cooperman *et al*, 2004); primary leukaemic T-ALL cells have a similar growth profile (Hirt *et al*, 1992). The wide range of growth rates among the cell line panel (23–442 h, Table 2) mirrors these reported values, indicating that they may be closer to the *in vivo* situation than is commonly believed for such cell lines. In accordance with published guidelines (Drexler and Matsuo, 1999; Drexler *et al*, 2003), we have validated the authenticity of these cell lines by genetic fingerprinting. Despite the fact that some were isolated up to 20 years ago, there was an impressive degree of concordance with the primary specimens from which they were derived, indicating a high degree of stability in long-term routine culture.

In this study, we have not directly tested the drug resistance profiles of the patient specimens from which our cell lines were derived. However, comparison with previously published data indicates that for each of the drugs tested here, except VCR, there are several cell lines within the panel that accurately reflect the sensitivity of leukaemic blasts tested *ex vivo*; for DEX, ARA-C, 6TG and DNR, the overlap between *in vitro* and *ex vivo* data is particularly strong. The lines included in this study have always been maintained without selection pressure (i.e. are grown without antibiotics and have had no drug exposure before MTT testing) and thus represent an ideal model system for the investigation of resistance mechanisms that may occur naturally *in vivo*; to this end, experiments are underway to examine the gene-expression profile of the panel by high-density oligonucleotide microarray. However, the consistent hypersensitivity of all cell lines to VCR may be an indication that *in vitro* studies of resistance for this drug should be interpreted with caution. The observation cannot be explained as an artefact of high proliferation rates in culture as many of these lines are extremely slow growing (Table 2), but may instead be a reflection of the well-documented inoculum effect whereby VCR cytotoxicity is increased at lower cell densities (Kobayashi *et al*, 1998). Alternatively, adaptation to an *in vitro* microenvironment may mitigate changes to cell architecture that render the cell more sensitive to the microtubule-blocking effects of VCR.

PER-145 was found to be highly resistant to MTX and had relatively high IC_50_ values for all of the drugs, except ASP for which it appears to have retained sensitivity (Table 2). This cell line has a complex karyotype (Table 1) and was derived from a patient who had received escalating doses of MTX (as per the Capizzi I schedule) in the 5 weeks immediately before his third relapse; the cell line was isolated from this third relapse specimen and has presumably undergone selection for MTX resistance *in vivo*. The patient subsequently relapsed for a fourth time after having received high-dose ARA-C; a description of an additional cell line isolated from this later time point and demonstrating resistance to ARA-C has previously been published (Kees, 1987; Kees *et al*, 1989a). The retention of ASP sensitivity in PER-145 may be related to the fact that this cell line carries the TEL-AML1 translocation (Kees *et al*, 2003), which has been linked to increased ASP sensitivity in ALL (Ramakers-van Woerden *et al*, 2000). Closer examination of the mechanism for MTX resistance in this cell line is currently underway.

In T-ALL cell lines, there was significant IC_50_ correlation between DNR, DOX, VCR and ARA-C, consistent with previous studies indicating mechanisms of cross-resistance for these drugs (Kaspers *et al*, 1998; Martin-Aragon *et al*, 2000; Lofgren *et al*, 2004). We found a positive correlation between glucocorticoid and ASP resistance, indicating that sensitivity to these unrelated compounds may also be influenced by common biological factors, possibly through alterations in apoptosis (Holleman *et al*, 2003). There was no correlation between the glucocorticoids and thiopurines, but between glucocorticoids and MTX there was in fact evidence of an inverse relationship. Lack of cross-resistance between MTX and glucocorticoids has previously been highlighted by a small study in ALL patient specimens (Hegge *et al*, 1999) but, to the best of our knowledge, a directly inverse relationship as indicated by the present data has not yet been described. It is interesting to speculate that this phenomenon may relate to differential expression of specific multidrug transporters, for example the coordinated upregulation of ABCG2 (breast cancer resistance protein or BCRP) and downregulation of ABCC1 (MRP1); such changes have recently been associated with reciprocal changes in the sensitivities to DEX and MTX in CEM cells (1.8-fold increase in resistance to MTX, and a 13-fold decrease in resistance to DEX (van der Heijden *et al*, 2004)). The ratio of these same drug transporters is also important for MTX pharmacodynamics in paediatric ALL (Kager *et al*, 2005). ABCG2 is a major transporter for MTX that can be directly inhibited by DEX and MPRED, but apparently not by prednisolone (Pavek *et al*, 2005). In the present study, MTX resistance was also negatively correlated with ASP resistance. The data therefore suggest that for patients who show resistance to glucocorticoids and ASP (e.g. at relapse), treatment with MTX may be increasingly relevant. Further work is required to confirm the observed reciprocity between these drugs but, if genuine, the finding has direct implications for the clinical setting and the design of protocols for relapsing patients.

## Figures and Tables

**Table 1:** Characterisation of ALL cell lines

**Cell line**	**Isolated**	**Phenotype**	**Specimen**	**Karyotype**	**Allelic concordance (%)**	**References**
PER-117	1984	T-ALL	R2	53,add(X)(q24),Y,t(1;11;9)(p13;p11;p22),+3,+7,+8,add(8)(q24.3),−9,t(12;18)(q24,q11),add(14)(q32),+13,+15,+19, +mar1,+mar2	88	1, 2
PER-255	1986	T-ALL	D	46,XY,t(7;10)(q32–q34;q24),t(9;12)(p22;p12–13)	94	1, 4, 5
PER-427	1990	T-ALL	D	46,XX	100	1
PER-487	1992	T-ALL	D	46,XY	100	1
PER-537	1995	T-ALL	D	47,XY,+8	100	1
PER-550	1996	T-ALL	R2	47,XY,add(1)(p36),t(1;8)(q?25;q?24),del(4)(q12),add(4)(p14),add(4)(q21),add(7)(q32),add(12)(q24),del(14)(q22q32), add(20)(q13),+mar	100	1
PER-604	2003	T-ALL	R1	46,XY,del(6)(q13q25),del(17)(p11) [16]	97	
PER-606	2003	T-ALL	R1	47,XY,del(6)(q21q25),add(7)(q3?2),+8,add(10)(q24),-11,add(13)(q34),add(17)(q2?1),+mar[24]	100	
PER-608	2003	T-ALL	D	46,XY	100	
PER-145	1985	Pre-B-ALL	R3	45,XY,der(3)t(3;?)(q12;?),der(4)t(4;?)(p15.2;?),t(5;17)(q15;p13),der(7)t(7;8)(p13–14;q13–21),-8,?t(9;20;9)(p13;p12;q34), der(12)t(12;?)(p13;?)	100	1, 3, 6
PER-278	1987	Pre-B-ALL	D	46,XY,der(9)t(1;9)(q23;p13),der(19)t(1;19)(q23;p13)	100	1, 7
PER-371	1988	Pre-B-ALL	D	46,XY,der(16)t(1;16)(q2?1;p13),der(19)t(1;19)(q?13;p13)/46,X,−Y,+?der(1)t(Y;1)(q12;?q21),add(11)(q21),der(19)t(1;19)	100	1
PER-377	1989	B-ALL	R1	46,XY,t(2;13)(p12;q34),del(7)(q11q21),?inv(14)(q11q24),der(17)t(8;17)(q11;p11),47,XY,idem,t(1;20)(q32;q13),+19	88	1, 8, 9
PER-485	1992	Infant Pre-B-ALL	R1	47,XX,der(4)t(4;11)(q21;q23),add(4)(p16),+6,del(7)(p14),add(8)(q24.3),der(9)inv(9)(p11q12)del(9)(p24), der(11)t(4;11)(q21;q23)	100	1
PER-490	1992	Infant Pre-B-ALL	D	46,XX,t(4;11)(q21;q23)/46,XX,t(4;11),dup(1)(q12q44)/46,XX,t(4;11),der(2)t(1;2)(q12;q37)	100	1
PER-495	1992	B-ALL	R1	46,XY,t(8;14)(q24;q32)	100	1
PER-607	2003	EBV	D	46,XY	100	

ALL=acute lymphoblastic leukaemia; EBV=Epstein–Barr virus-transformed cell line.

Cell lines were derived from diagnosis (D), or first/second/third (R1, R2, R3) relapse specimens. References: (1) Kees *et al* (2003); (2) Kees *et al* (1987); (3) Kees *et al* (1989a); (4) Kees *et al* (1989b); (5) Kennedy *et al* (1991); (6) Kees (1987); (7) Kees *et al* (1990); (8) Kees *et al* (1995); (9) Whitman *et al* (2001).

**Table 2:** Growth and drug resistance profile of ALL cell lines

**Cell line**	**Phenotype**	**Specimen**	**DT (h)**	**ARA-C**	**6MP**	**6TG**	**MTX**	**DEX**	**MPRED**	**DNR**	**DOX**	**ASP**	**VCR**
ALL-SIL	T-ALL	Relapse	64	0.0303	0.416	0.652	0.00649	11.620	137.31	0.00933	0.09500	0.00007	0.00020
CEM	T-ALL	Relapse	23	0.0939	1.240	0.613	0.00599	500	419.88	0.20400	0.42900	1.99695	0.00213
DU528	T-ALL	Diagnosis	40	0.0123	65.800	1.845	0.0101	500	190.16	0.05300	0.18800	0.00003	0.00308
HSB2	T-ALL	Diagnosis	40	0.0020	0.283	0.141	0.0055	2	276.98	0.00092	0.00072	0.04390	0.00013
JURKAT	T-ALL	Relapse	26	0.0153	0.410	0.334	0.00784	500	500	0.01400	0.03270	1.25424	0.00168
MOLT4	T-ALL	Relapse	33	0.0129	0.454	0.348	0.00671	500	356.04	0.00319	0.01360	0.09690	0.00078
PER-117	T-ALL	Relapse	56	0.0059	0.042	0.049	0.00539	32.440	85.71	0.00435	0.01100	0.03022	0.00022
PER-255	T-ALL	Diagnosis	66	0.0033	0.223	0.060	0.00683	0.003	0.05	0.00163	0.00475	0.00004	0.00016
PER-427	T-ALL	Diagnosis	209	0.2810	22.500	50.800	0.00702	51.990	0.19	0.01110	0.05460	0.43701	0.00493
PER-487	T-ALL	Diagnosis	363	0.0264	15.100	3.175	0.0111	189.080	14.45	0.01060	0.05410	0.00008	0.00141
PER-537	T-ALL	Diagnosis	223	0.0172	22	1.072	0.0339	167.020	9.86	0.00325	0.01340	0.00016	0.00036
PER-550	T-ALL	Relapse	252	0.0030	3.550	0.440	0.0333	0.002	0.02	0.00163	0.00956	0.00003	0.00025
PER-604	T-ALL	Relapse	181	0.0059	16	1.572	0.0866	0.117	0.17	0.00428	0.03610	0.00003	0.00080
PER-606	T-ALL	Relapse	442	0.0008	8.460	0.758	0.0678	0.001	0.02	0.00216	0.00762	0.00003	0.00017
PER-608	T-ALL	Diagnosis	260	0.0073	6.670	0.142	0.0123	0.003	0.06	0.00128	0.00405	0.00004	0.00033
			Median	0.0123	3.550	0.613	0.0078	32.440	14.45	0.00430	0.01360	0.00010	0.00040
PER-145	Pre-B-ALL	Relapse	108	0.0572	173.965	10.940	725.1374	500	84.75	0.04880	0.05436	0.00007	0.00161
PER-278	Pre-B-ALL	Diagnosis	104	0.0073	47.754	1.795	0.00929	0.072	1.08	0.00245	0.00904	0.28644	0.00080
PER-371	Pre-B-ALL	Diagnosis	69	0.0040	39.708	71.600	0.01466	0.003	0.06	0.00600	0.01174	0.95612	0.00089
PER-377	B-ALL	Relapse	66	0.0211	0.829	0.360	0.00546	0.0329	26.13	0.01230	0.08807	1.20029	0.01150
PER-490	Infant Pre-B-ALL	Diagnosis	97	0.0279	97.495	3.200	0.00602	0.002	0.01	0.00669	0.02071	0.00003	0.00073
PER-495	B-ALL	Relapse	53	0.0034	0.080	0.037	0.00637	304.170	180.12	0.00419	0.01045	0.01631	0.00059
PER-485	Infant Pre-B-ALL	Relapse	53	2.5000	0.321	0.338	0.00245	500	500	0.00358	0.15423	0.19488	0.00030
			Median	0.0211	39.7077	1.7950	0.0064	0.0719	26.1250	0.0060	0.0207	0.1949	0.0008
PER-607	EBV	Diagnosis	216	0.9992	62.198	13.580	0.01312	307	301.46	0.01110	0.07281	0.31872	0.00230

ALL=acute lymphoblastic leukaemia; ARA-C=cytosine arabinoside; ASP=L-asparaginase; DEX=dexamethasone; DNR=daunorubicin; DOX=doxorubicin; DT=doubling time; EBV=Epstein–Barr virus-transformed cell line; 6MP=6-mercaptopurine; MTX=methotrexate; 6TG=6-thioguanine; MPRED=methylprednisolone; VCR=vincristine.

IC_50_ units are *μ*g ml^−1^, except for ASP (IU ml^−1^).

**Table 3:** Details of *ex vivo* resistance studies used for comparison with *in vitro* data

**Drug**	**No. of studies**	**No. of diagnosis specimens**	**No. of relapse specimens**	**Total No. of specimens**	**References**
DEX	7	737	248	985	1, 2, 5, 8, 9, 11, 13
MPRED	1	13	6	19	9
VCR	9	1137	275	1412	1, 2, 5–8, 11–13
ARA-C	9	995	331	1326	1–3, 5, 8, 10–13
6MP	5	764	220	984	1, 2, 5, 8, 11
6TG	9	973	291	1264	1–3, 5, 7, 8, 11–13
DNR	8	1126	278	1404	1, 2, 5, 7, 8, 11–13
DOX	5	580	257	837	1, 2, 5, 8, 11
ASP	7	1013	275	1288	1, 2, 4, 5, 8, 11–12

ARA-C=cytosine arabinoside; ASP=L-asparaginase; DEX=dexamethasone; DNR=daunorubicin; DOX=doxorubicin; 6MP=6-mercaptopurine; 6TG=6-thioguanine; MPRED=methylprednisolone; VCR=vincristine.

References: (1) Klumper *et al* (1995); (2) Pieters *et al* (1998); (3) Duyn *et al* (1999); (4) Fine *et al* (2005); (5) Kaspers *et al* (2005); (6) Mihal *et al* (2004); (7) Pieters *et al* (1991); (8) Styczynski *et al* (2000); (9) Styczynski *et al* (2002); (10) Styczynski *et al* (2005); (11) Zwaan *et al* (2000); (12) Pieters *et al* (1990); (13) Steinbach *et al* (2005).

**Table 4:**
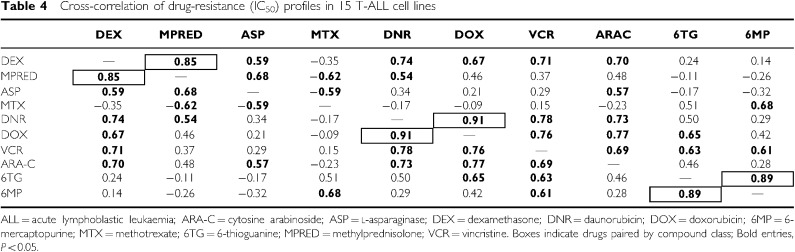
Cross-correlation of drug-resistance (IC_50_) profiles in 15 T-ALL cell lines
