# Supplementary material for: Authenticity and drug resistance in a panel of acute lymphoblastic leukaemia cell lines
Source: Br J Cancer. 2006 Nov 21;95(11):1537–44. doi: 10.1038/sj.bjc.6603447 (PMC2360743; doi:10.1038/sj.bjc.6603447)
Supplement: Supplementary Table 1 [file 95-6603447x1.doc]

Supplementary Table 1. DNA Fingerprint Comparison of Cell Lines and Patient Specimens

| **Cell Lines** | | | | | | | | | | | | | | | | | |
| --- | --- | --- | --- | --- | --- | --- | --- | --- | --- | --- | --- | --- | --- | --- | --- | --- | --- |
| **LOCUS** | **PER-117** | **PER-255** | **PER-427** | **PER-487** | **PER-537** | **PER-550** | **PER-604** | **PER-606** | **PER-608** | **PER-145** | **PER-278** | **PER-371** | **PER-377** | **PER-485** | **PER-490** | **PER-495** | **PER-607** |
| CSF1PO | 10,13 | 10,11 | 12,12 | 9,13 | 10,10 | 10,12 | **11,(12),13** | 10,12 | 10,11 | 11,13 | 11,12 | 12,13 | 11,12 | 11,11 | 11,11 | 12,12 | 10,12 |
| D7S820 | 9,13 | 11,11 | 11,12 | 9,11 | 8,10 | 10,13 | 8,11 | 10,13 | 8,10 | 10,12 | 11,12 | 8,10 | **11,12** | 10,11 | 10,11 | 8,9 | 8,9 |
| D21S11 | **31,32.2** | 30,31.2 | 29,29 | 29,30 | 28,30 | 28,28 | 30,31.2 | 28,28 | 29,30 | 30,33.2 | 29,31 | 31.2,31.2 | 31.2,32 | 30.2,31.2 | 31,33.2 | 29,30 | 30,31.2 |
| D8S1179 | **13,13** | **14,14** | 12,14 | 8,13 | 13,14 | 12,13 | 12,13 | 12,13 | 14,14 | 9,11 | 11,12 | 10,14 | 12,14 | 13,15 | 12,14 | 11,12 | 13,16 |
| D2S1338 | 16,17 | 17,17 | 21,26 | 17,20 | 20,26 | 20,24 | 24,25 | 20,24 | 16,19 | 16,19 | 23,25 | 23,24 | 19,21 | 24,25 | 23,26 | 17,17 | 19,20 |
| D16S539 | **11,13** | 9,12 | 12,12 | 10,12 | 12,12 | 12,13 | 10,11 | 12,13 | 9,10 | 12,13 | 13,13 | 11,13 | 11,11 | 11,11 | 11,12 | 12,14 | 11,11 |
| D13S317 | **8,(11),12** | 12,13 | 11,12 | 13,13 | 8,14 | 10,11 | 11,12 | 10,11 | 13,13 | 8,9 | 8,9 | 10,12 | **12,12** | 8,12 | 11,12 | 11,12 | 12,12 |
| TH01 | 8,9.3 | 9,9.3 | 9,9.3 | 6,8 | 6,8 | 9.3,9.3 | 6,9 | 9.3,9.3 | 7,9.3 | 6,9.3 | 6,8 | 6,6 | 6,9.3 | 6,9 | 6,9.3 | 8,9 | 6,7 |
| D3S1358 | 15,(17) | 15,18 | 14,15 | 16,17 | 15,17 | 14,17 | 15,(17) | 14,17 | 15,17 | 18,18 | 18,18 | 15,15 | 16,16 | 16,18 | 15,17 | 19,19 | 16,16 |
| D18S51 | 12,13 | **19,19** | 14,18 | 12,17 | 11,15 | 10,12 | 11,12,17 | 10,12 | 12,17 | 14,16 | 14,15 | 10,17 | 14,17 | 12,16 | 12,17 | 14,17 | 15,22 |
| TPOX | 8,11 | 8,8 | 11,12 | 8,11 | 9,11 | 11,11 | 8,9 | 11,11 | 8,8 | 9,11 | 8,11 | 10,11 | 11,12 | 9,9 | 8,8 | 8,9 | 9,11 |
| vWA | 14,17 | 16,17 | 15,19 | 14,18 | 17,18 | 15,15 | 17,(18) | 15,15 | 16,18 | 17,17 | 14,19 | 15,16 | 14,17 | 15,16 | 15,17 | 15,16 | 16,17 |
| D19S433 | 14,14 | 13,14 | 12,13 | 14,14 | 13,14 | 13,14 | 14,16.2 | 13,14 | 14,15 | 16,6 | 13,15 | 13,14 | 13,14 | 14,16.2 | 13,15 | 14,15 | 13,13 |
| FGA | 23,24 | 23,24 | 21,24 | 23,24 | 21,21 | 21,25 | 20,21 | 21,25 | 21,22 | 19,20 | 24,27 | 23,25 | 22,23 | 20,24 | 22,24 | 22,26 | 22,26 |
| D5S818 | 11,(12) | 12,13 | 12,12 | 9,13 | 11,11 | 11,12 | 11,11 | 11,12 | 11,12 | 10,11 | 12,13 | 11,12 | 12,12 | 12,12 | 11,12 | 11,12 | 11,11 |
| Amelogenin | X(Y) | XY | XX | XY | XY | XY | XY | XY | X(Y) | XY | XY | XY | **XX** | XX | XX | XY | XY |
|  |  |  |  |  |  |  |  |  |  |  |  |  |  |  |  |  |  |
| **Patient Specimens** | | | | | | | | | | | | | | | | | |
| CSF1PO | 10,(12),13 | 10,11 | 12,12 | 9,13 | 10,10 | 10,12 | 11,13 | 10,12 | 10,11 | 11,13 | 11,12 | 12,13 | 11,12 | 11,11 | 11,11 | 12,12 | 10,12 |
| D7S820 | 9,13 | 11,11 | 11,12 | 9,11 | 8,10 | 10,13 | 8,11 | 10,13 | 8,10 | 10,12 | 11,12 | 8,10 | 12,13 | 10,11 | 10,11 | 8,9 | 8,9 |
| D21S11 | 30,32.2 | 30,31.2 | 29,29 | 29,30 | 28,30 | 28,28 | 30,31.2 | 28,28 | 29,30 | 30,33.2 | 29,31 | 31.2,31.2 | 31.2,32 | 30.2,31.2 | 31,33.2 | 29,30 | 30,31.2 |
| D8S1179 | 13,14,(15) | 11,14 | 12,14 | 8,13 | 13,14 | 12,13 | 12,13 | 12,13 | (12),14 | 9,11 | 11,12 | 10,14 | 12,14 | 13,15 | 12,14 | 11,12 | 13,16 |
| D2S1338 | 16,17 | 17,17 | 21,26 | 17,20 | 20,26 | 20,24 | 24,25 | 20,24 | 16,19 | 16,19 | 23,25 | 23,24 | 19,21 | 24,25 | 23,26 | 17,17 | 19,20 |
| D16S539 | 12,13 | 9,12 | 12,12 | 10,12 | 12,12 | 12,13 | 10,11 | 12,13 | 9,10 | 12,13 | 13,13 | 11,13 | 11,(12) | 11,11 | 11,12 | 12,14 | 11,11 |
| D13S317 | 8,12 | 12,13 | 11,12 | 13,13 | 8,14 | 10,11 | 11,12 | 10,11 | 13,13 | 8,9 | 8,9 | 10,12 | 11,11 | 8,12 | 11,12 | 11,12 | 12,12 |
| TH01 | (6),8,9.3 | 9,9.3 | 9,9.3 | 6,8 | 6,8 | 9.3,9.3 | 6,9 | 9.3,9.3 | 7,9.3 | 6,9.3 | 6,8 | 6,6 | 6,9.3 | 6,9 | 6,9.3 | 8,9 | 6,7 |
| D3S1358 | (14),15,17 | 15,18 | 14,15 | 16,17 | 15,17 | 14,17 | 15,(17) | 14,17 | 15,17 | 18,18 | 18,18 | 15,15 | 16,16 | 16,18 | 15,17 | 19,19 | 16,16 |
| D18S51 | 12,13 | 18,19 | 14,18 | 12,17 | 11,15 | 10,12 | 11,12,17 | 10,12 | 12,17 | 14,16 | 14,15 | 10,17 | 14,17 | 12,16 | 12,17 | 14,17 | 15,22 |
| TPOX | 8,11 | 8,8 | 11,12 | 8,11 | 9,11 | 11,11 | 8,9 | 11,11 | 8,8 | 9,11 | 8,11 | 10,11 | 11,12 | 9,9 | 8,8 | 8,9 | 9,11 |
| vWA | 14,17,(18) | 16,17 | 15,19 | 14,18 | 17,18 | 15,15 | 17,(18) | 15,15 | 16,18 | 17,(20) | 14,19 | 15,16 | 14,17 | 15,16 | 15,17 | 15,16 | 16,17 |
| D19S433 | 14,(15) | 13,14 | 12,13 | 14,14 | 13,14 | 13,14 | 14,16.2 | 13,14 | 14,15 | 16,16 | 13,15 | 13,14 | 13,14 | 14,16.2 | 13,15 | 14,15 | 13,13 |
| FGA | 23,24 | 23,24 | 21,24 | 23,24 | 21,21 | 21,25 | 20,21 | 21,25 | 21,22 | 19,20 | 24,27 | 23,25 | 22,23 | 20,24 | 22,24 | 22,26 | 22,26 |
| D5S818 | 11,(12) | 12,13 | 12,12 | 9,13 | 11,11 | 11,12 | 11,11 | 11,12 | 11,12 | 10,11 | 12,13 | 11,12 | 12,12 | 12,12 | 11,12 | 11,12 | 11,11 |
| Amelogenin | XY | XY | XX | XY | XY | XY | XY | XY | XY | XY | XY | XY | XY | XX | XX | XY | XY |

Since bone marrow specimens may provide data from multiple leukemic clones, allelic profiles were compared using cell lines as reference. Allelic concordance was calculated as the percentage of cell line alleles that were detectable in the original patient specimen. Shaded boxes indicate loci where this was not the case. Minor alleles detected at lower concentrations are indicated in brackets.
